# Rare Forms of Cardiac Amyloidosis: Diagnostic Clues and Phenotype in Apo AI and AIV Amyloidosis

**DOI:** 10.1161/CIRCIMAGING.123.015259

**Published:** 2023-07-11

**Authors:** Adam Ioannou, Aldostefano Porcari, Rishi K. Patel, Yousuf Razvi, Giulio Sinigiani, Ana Martinez-Naharro, Lucia Venneri, James Moon, Muhammad U. Rauf, Helen Lachmann, Ashutosh Wechelakar, Philip N. Hawkins, Julian D. Gillmore, Marianna Fontana

**Affiliations:** 1 National Amyloidosis Centre, University College London, Royal Free Campus, United Kingdom (A.I., A.P., R.K.P., Y.R., A.M.-N., L.V., M.U.R., H.L., A.W., P.N.H., J.D.G., M.F.).; 2 Center for Diagnosis and Treatment of Cardiomyopathies, Cardiovascular Department, Azienda Sanitaria Universitaria Giuliano-Isontina (ASUGI), University of Trieste, European Reference Network for Rare, Low Prevalence and Complex Diseases of the Heart-ERN GUARD-Heart, Italy (A.P.).; 3 Department of Cardiac, Thoracic and Vascular Sciences and Public Health, University of Padua, Italy (G.S.).; 4 St Bartholomew’s Hospital, W Smithfield, London, United Kingdom (J.M.).

**Keywords:** amyloidosis, hereditary, transthyretin-related, apolipoprotein, phenotype, prognosis

## Abstract

Apo AI amyloidosis (AApoAI) and Apo AIV amyloidosis (AApoAIV) are rare but increasingly recognized causes of cardiac amyloidosis (CA). We sought to define the cardiac phenotype in AApoAI and AApoAIV using multimodality imaging.

**METHODS:**

We identified all patients with AApoAI and AApoAIV assessed at our center between 2000 and 2021, and 2 cohorts of patients with immunoglobulin light-chain amyloidosis (AL) and transthyretin amyloidosis matched for age, sex, and cardiac involvement.

**RESULTS:**

Forty-five patients had AApoAI, 13 (29%) of whom had cardiac involvement, 32 (71%) renal involvement, 28 (62%) splenic involvement, 27 (60%) hepatic involvement, and 7 (16%) laryngeal involvement. AApoAI-CA commonly presented with heart failure (n=8, 62%) or dysphonia (n=7, 54%). The Arg173Pro variant universally caused cardiac and laryngeal involvement (n=7, 100%). AApoAI-CA was associated with right-sided involvement, with a thicker right ventricular free wall (8.6±1.9 versus 6.3±1.3 mm versus 7.7±1.2 mm, *P*=0.004), greater incidence of tricuspid stenosis (4 [31%] versus 0 [0%] versus 0 [0%], *P*=0.012) and tricuspid regurgitation (6 [46%] versus 1 [8%] versus 2 [15%], *P*=0.048) than AL-CA and transthyretin CA. Twenty-one patients had AApoAIV, and cardiac involvement was more common than in AApoAI (15 [71%] versus 13 [29%], *P*=0.001). AApoAIV-CA most commonly presented with heart failure (n=12, 80%), and a lower median estimated glomerular filtration rate than AL-CA and transthyretin CA (36 mL/[min·1.73 m²] versus 65 mL/[min·1.73 m²] versus 63 mL/[min·1.73 m²], *P*<0.001). All AApoAIV-CA patients had classical CA features on echocardiography/cardiac magnetic resonance, including an apical-sparing strain pattern, which was less common in AApoAI-CA (15 [100%] versus 7 [54%], *P*=0.003), whereas cardiac uptake on bone scintigraphy was less common in AApoAIV-CA than AApoAI-CA (all grade 1) (14% versus 82%, *P*<0.001). Patients with AApoAI and AApoAIV had a good prognosis (median survival >172 and >30 months, respectively), and a lower risk of mortality than matched patients with AL-amyloidosis (AL versus AApoAI: hazard ratio, 4.54 [95% CI, 2.02–10.14]; *P*<0.001; AL versus AApoAIV: hazard ratio, 3.07 [95% CI, 1.27–7.44]; *P*=0.013).

**CONCLUSIONS:**

Dysphonia, multisystem involvement, or right-sided cardiac disease should raise suspicion of AApoAI-CA. AApoAIV-CA presents most commonly with heart failure and always displays classical CA imaging features, mimicking common forms of CA. Both AApoAI and AApoAIV are associated with a good prognosis and a lower risk of mortality than matched patients with AL-amyloidosis.

**CLINICAL PERSPECTIVE**

Apo AI amyloidosis and Apo AIV amyloidosis are rare, but increasingly recognized causes of cardiac amyloidosis (CA). Apo AI CA is associated with a strong family history of amyloidosis, multisystemic disease, laryngeal involvement presenting with dysphonia, and right-sided cardiac disease on cardiac imaging. Apo AIV CA commonly presents with heart failure symptoms, and always displays characteristic features of CA on both echocardiography and cardiac magnetic resonance imaging, hence mimicking more common forms of CA. Appreciation of the unique characteristic features of Apo AI amyloidosis but also the capability of Apo AIV amyloidosis to mimic common forms of CA is urgently needed in clinical practice to reduce the risk of misdiagnosis.

Systemic amyloidosis describes a heterogeneous group of diseases characterized by deposition of misfolded protein fibrils within extracellular space, leading to organ dysfunction.^1^ Cardiac amyloidosis (CA) occurs when the deposition of amyloid fibrils causes distortion of the myocardial contractile elements. It is characterized by biventricular wall thickening and stiffening of the myocardium, which ultimately leads to progressive heart failure. Although amyloidosis is a multisystem disease, cardiac involvement remains the leading cause of mortality.^2,3^

The vast majority of CA cases are represented by misfolded immunoglobulin light-chain (immunoglobulin light-chain amyloidosis [AL]) and transthyretin (transthyretin amyloidosis [ATTR]) proteins.^3–5^ Less common, although increasingly recognized causes include Apo AI amyloidosis (AApoAI) and Apo AIV amyloidosis (AApoAIV).^6,7^ The treatment is very different in different types of CA ranging from chemotherapy in systemic AL-amyloidosis to transthyretin stabilizers^8^ and gene silencers in ATTR.^9^ It is, therefore, crucial to raise awareness of rare forms of CA, which include AApoAI and AApoAIV, to avoid misdiagnosis and subsequent inappropriate treatment.

ApoAI is a physiological plasma protein of 28 kDa synthesized by the liver and small intestine. It is the major protein component of high-density lipoprotein particles in the plasma and is involved in cholesterol transport. Wild-type ApoAI-amyloid has been identified in atherosclerotic plaques and is thought to form secondary to the atherosclerotic inflammatory process. Wild-type ApoAI-amyloid fibrils remain localized within the atherosclerotic plaque and are not associated with systemic disease.^10^ In contrast, the misfolding that occurs secondary to pathogenic mutations in the *ApoAI*-gene promotes deposition in various organs throughout the body, resulting in disruption of organ structure and function, and development of a systemic disease process known as AApoAI.^11^

ApoAIV is a 46 kDa protein synthesized predominantly in the small intestine and plays a role in the absorption, transportation, and metabolism of lipids, but unlike AApoAI, the disease is not hereditary and mechanisms that promote the development of AApoAIV remain obscure.^12^ Although the more common forms of CA (AL and ATTR) have been well characterized, data on the cardiac phenotype of patients with AApoAI and AApoAIV is sparse, with the majority of available literature being case reports and small case series.^6,7^

The aim of this study was to define the clinical phenotype in patients with AApoAI and AApoAIV-CA using extensive characterization comprising biomarkers and multimodality cardiac imaging.

## METHODS

The data that support the findings of this study are available from the corresponding author upon reasonable request.

### Patient Samples

We performed a retrospective search of the National Amyloidosis Center database, to identify all patients with a confirmed diagnosis of AL, ATTR, AApoAI, and AApoAIV amyloidosis who underwent a cardiac assessment, between 2000 and 2021. This study included 66 patients with AL, 66 patients with ATTR, 45 patients with AApoAI, and 21 patients with AApoAIV amyloidosis.

Diagnoses were confirmed based on validated diagnostic criteria. All biopsies were stained with Congo red dye and viewed under crossed polarized light. The definitive amyloid fibril type was established by immunohistochemical staining of amyloid deposits using monospecific antibodies or by microdissection of amyloid deposits and proteomic analysis. The diagnosis of AL-amyloidosis was confirmed by central review of histology. The diagnosis of ATTR-amyloidosis was based on either the presence of polyneuropathy, biopsy proof of ATTR-amyloid, and *TTR*-gene variant or the presence of heart failure symptoms, characteristic cardiac imaging, and either biopsy proof of ATTR-amyloid, or grade 2 to 3 uptake on bone scintigraphy in the absence of biochemical evidence of a plasma cell dyscrasia. AApoAI was confirmed by identification of a pathogenic *ApoAI*-gene variant with either histological proof of AApoAI-amyloid organ deposition or imaging evidence of organ deposition. AApoAIV was confirmed by histological and proteomic proof of AApoAIV tissue infiltration.^13^ Of note, data from 36 patients had been included within a prior article from our center, but the current study includes updated analysis.^14^ Patients were managed in accordance with the Declaration of Helsinki and provided written informed consent for analysis and publication of their data.

Cardiac amyloid infiltration was ascertained using a combination of bone scintigraphy, echocardiography, and cardiac magnetic resonance (CMR) imaging, and in some cases corroborated with direct endomyocardial biopsy proof of cardiac infiltration. Intensity of cardiac uptake on the planar bone scintigraphy was categorized as 0–3 according to the grading system described by Perugini et al,^15^ and right ventricular (RV) involvement was defined as RV uptake on single-photon emission computed tomography. Echocardiograms were described as suggestive of CA or characteristic of CA if the maximal wall thickness was >12 mm and a total score of 2 to 7 points or ≥8 points respectively using the following scoring system: relative wall thickness >0.6=3 points, E/e′ >11=1 point, tricuspid annular plane systolic excursion <19 mm=2 points, longitudinal strain >−13%=1 point, systolic apex-to-base ratio >2.9=3 points.^13,16^ RV involvement was defined as an RV free wall thickness >5 mm, and valvular regurgitation deemed significant if at least moderate or severe. CMR images were characterized using the following grading system: no features of CA (normal left ventricular [LV] mass, no late gadolinium enhancement [LGE], and normal extracellular volume [ECV]), early cardiac amyloid infiltration (normal LV mass, raised ECV or subendocardial LGE), and characteristic of CA (increased LV mass, diffuse subendocardial or transmural LGE, altered gadolinium kinetics, and raised ECV). RV involvement was defined as subendocardial or transmural RV LGE.

Technical aspects related to the acquisition of bone scintigraphy, serum amyloid P component scintigraphy, echocardiographic, and CMR images are described in the Supplemental Material.

### Statistical Analysis

Statistical analysis was performed using Stata (StataCorp. 2021. Stata Statistical Software: Release 17. College Station, TX: StataCorp LLC). All continuous variables were tested for normal distribution (Shapiro-Wilk test) and presented as mean±SD or median (interquartile range), other than NT-proBNP (N-terminal pro-B-type natriuretic peptide) which was natural log-transformed for parametric testing. One-way ANOVA if the data were normally distributed in each group was used to compare means, or its nonparametric equivalent (Kruskal-Wallis test) was used to compare the distribution of multiple groups. A significant result was followed by a post hoc Bonferroni corrected pairwise comparison to establish where differences lay. Categorical data are presented as absolute numbers (n) and frequencies (%) and compared using the χ^2^ test or Fisher exact test as appropriate. Case-control matching was used to match patients diagnosed with AApoAI and AApoAIV for age, sex, and presence or absence of cardiac involvement, to corresponding patients with AL- and ATTR-amyloidosis in a 1:1 ratio.

All mortality data were obtained via the UK Office of National Statistics. The mortality end point was defined as time to death from baseline for all deceased patients and time to censor date (January 27, 2023) from baseline among the remainder. Survival was evaluated using Cox proportional hazards regression analysis, providing estimated hazard ratios (HRs) with 95% CIs. The proportional hazards assumption was checked and confirmed. Kaplan-Meier curves were constructed, with statistical significance being assessed with a log-rank test. Statistical significance was defined as *P*<0.05.

## RESULTS

We identified 4364 patients with AL-amyloidosis, of which 2730 (62.6%) had cardiac involvement, 2057 patients with ATTR-amyloidosis, of which 1984 (96.5%) had cardiac involvement, 45 patients with AApoAI, of which 13 (28.9%) had cardiac involvement and 21 patients with AApoAIV, of which 15 (71.4%) had cardiac involvement. The patients with AApoAI and AApoAIV made up 1.0% of cases assessed at the National Amyloidosis Center during the study period.

### Apo AI Amyloidosis

#### Baseline Characteristics

We identified 45 patients with AApoAI (mean age, 51.6±13.4 years; male, 24 [53.3%]) who had the following *ApoAI*-gene variants: Gly26Arg=22, Arg173Pro=7, Leu60Arg=3, Phe71Tyr=3, His155Met=2, Leu90Pro=2, Trp50Arg=2, E70_W72=1, Gln172Pro=1, Glu34Lys=1, IL60Arg=1. Biopsy and ethnicity data are presented in the Supplemental Material.

The most common reason for referral was for investigation of chronic kidney disease (n=21, 46.7%), followed by genetic screen for a positive family history (n=9, 20.0%), symptoms of heart failure, and local cardiac imaging suggestive of CA (n=8, 17.8%), and amyloid infiltration seen on a biopsy (n=7, 15.6%). On taking a detailed medical history, 26 (57.8%) patients reported a family history of amyloidosis, and 5 (11.1%) reported symptoms of peripheral neuropathy. Multisystemic disease was common in patients with AApoAI. Renal involvement was most common (n=32, 71.1%), followed by splenic involvement (n=28, 62.2%), hepatic involvement (n=27, 60.0%), cardiac involvement (n=13, 28.9%), and laryngeal involvement (n=7, 15.6%).

#### Cardiac Phenotype

A total of 13 (28.9%) patients had cardiac involvement (Arg173Pro=7, Leu60Arg=3, Gln172Pro=1, IL60Arg=1, Leu90Pro=1). Patients with cardiac involvement were referred with heart failure symptoms and imaging suggestive of CA (n=8, 61.5%) or amyloid infiltration identified on biopsy (n=5, 38.5%). The Arg173Pro variant was always associated with CA and also always associated with laryngeal involvement (n=7, 100.0%), with all patients reporting dysphonia, 4 of whom had a laryngeal biopsy confirming amyloid infiltration. The Arg173Pro variant was also associated with a petechial rash in some patients (n=4, 57.1%). Visceral organ involvement was common in those with cardiac involvement, 6 (46.2%) had renal involvement, 5 (38.5%) had liver involvement, and 4 (30.8%) had spleen involvement.

The baseline 12-lead ECG demonstrated sinus rhythm (n=9) in the majority of patients (all had a normal PR interval), 2 had atrial fibrillation, and 2 had a ventricular paced rhythm. Two patients had QRS complexes that met the low voltage criteria (QRS amplitude <0.5 mV in the limb leads and <1.0 mV in the precordial leads). All the remaining patients had a normal QRS amplitude (limb leads: 0.71±0.29mV; precordial leads: 1.39±0.37mV). The most common QRS pattern was Q waves in leads V_1_ to V_3_ (n=4), followed by right bundle branch block (n=2).

At diagnosis, all patients with AApoAI-CA underwent echocardiographic assessment. All patients had prominent RV free wall thickening (n=13, 100.0%), but only 5 (38.5%) patients had an echocardiogram characteristic of CA and 8 (61.5%) had an echocardiogram suggestive of CA. When compared with matched patients, those with AApoAI-CA less commonly had an apical sparing strain pattern than AL-CA and ATTR-CA (7 [53.8%] versus 11 [84.6%] versus 12 [92.3%], *P*=0.045).

AApoAI-CA caused prominent valvular thickening with thickening of the leaflets and subvalvular apparatus, resulting in subsequent valvular dysfunction. This was most marked on assessment of the tricuspid valve, whereby thickening was associated with tethering of the septal leaflet (n=11, 84.6%), and resulted in significant tricuspid regurgitation (moderate=4, severe=2; 4 had mixed valve disease with concomitant tricuspid stenosis) and the majority had a raised pulmonary artery systolic pressure (>35 mm Hg; n=11, 84.6%). Mitral valve leaflet thickening was associated with tethering of the posterior leaflet (n=7, 53.8%), but this resulted in moderate regurgitation in only 1 patient.

When compared with matched patients, those with AApoAI-CA had a thicker RV free wall (8.6±1.9 mm versus 6.3±1.3 mm versus 7.7±1.2 mm, *P*=0.004), greater incidence of tricuspid stenosis (4 [30.8%] versus 0 [0.0%] versus 0 [0.0%], *P*=0.012), greater incidence of tricuspid regurgitation (6 [46.2%] versus 1 [7.7%] versus 2 [15.4%], *P*=0.048), and greater incidence of raised pulmonary artery systolic pressure (11 [84.6%] versus 5 [38.5%] versus 6 [46.2%], *P*=0.039) than AL-CA and ATTR-CA.

**Figure 1.**
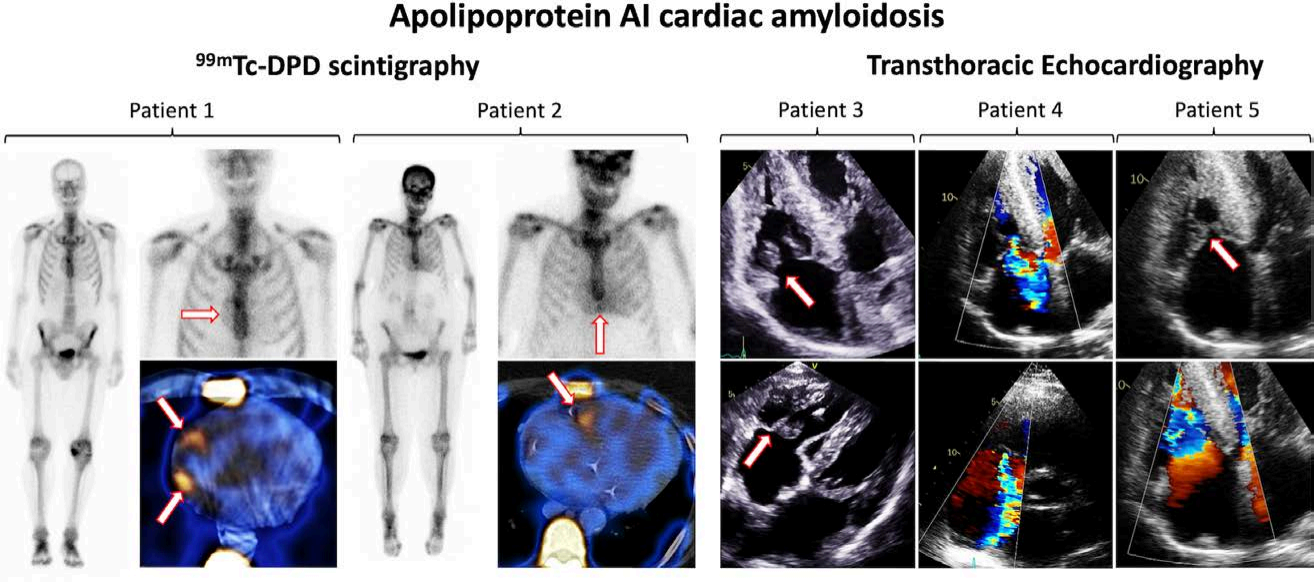
**^99m^Technetium labeled 3,3-diphosphono-1,2-propanodicarboxylic acid (^99m^Tc-DPD) scintigraphy and echocardiographic images of patients with Apo AI cardiac amyloidosis.** Patient 1=^99m^Tc-DPD scintigraphy and single-photon emission computed tomography (SPECT) images demonstrating grade 1 cardiac uptake with prominent right atrial uptake (arrows). Patient 2=^99m^Tc-DPD scintigraphy and SPECT images demonstrating grade 1 cardiac uptake with prominent right ventricular uptake (arrows). Patient 3=Transthoracic echocardiography demonstrating marked thickening of the tricuspid valve leaflets (arrows) and subvalvular apparatus and biventricular hypertrophy. Patient 4=Transthoracic echocardiography demonstrating severe tricuspid regurgitation. Patient 5=Transthoracic echocardiography demonstrating tricuspid stenosis (arrows), with turbulent forward flow through the tricuspid valve, biatrial enlargement, and biventricular hypertrophy.

At diagnosis 11 patients with AApoAI-CA underwent ^99m^Technetium labeled 3,3-diphosphono-1,2-propanodicarboxylic acid (^99m^Tc-DPD) scintigraphy, which demonstrated no cardiac uptake in 4 patients and grade 1 myocardial radiotracer uptake in 7 patients. Of the 4 patients who had no cardiac uptake at diagnosis, 2 had repeat ^99m^Tc-DPD scintigraphy at 1 and 7 years that demonstrated grade 1 myocardial radiotracer uptake; whereas 2 patients with grade 1 uptake at diagnosis had repeat scans at 1-year which did not demonstrate any changes. RV or right atrial (RA) uptake was observed in 7 out of 9 (77.8%) patients with cardiac radiotracer uptake (Figure 1). When compared with matched patients with ATTR-CA (all of whom demonstrated grade 2 to 3 cardiac uptake), those with AApoAI-CA more commonly demonstrated absent or grade 1 cardiac uptake (11 [100.0%] versus 0 [0.0%], *P*<0.001). Only 3 matched patients with AL-CA underwent ^99m^Tc-DPD scintigraphy, and therefore, no meaningful comparisons could be made.

**Figure 2.**
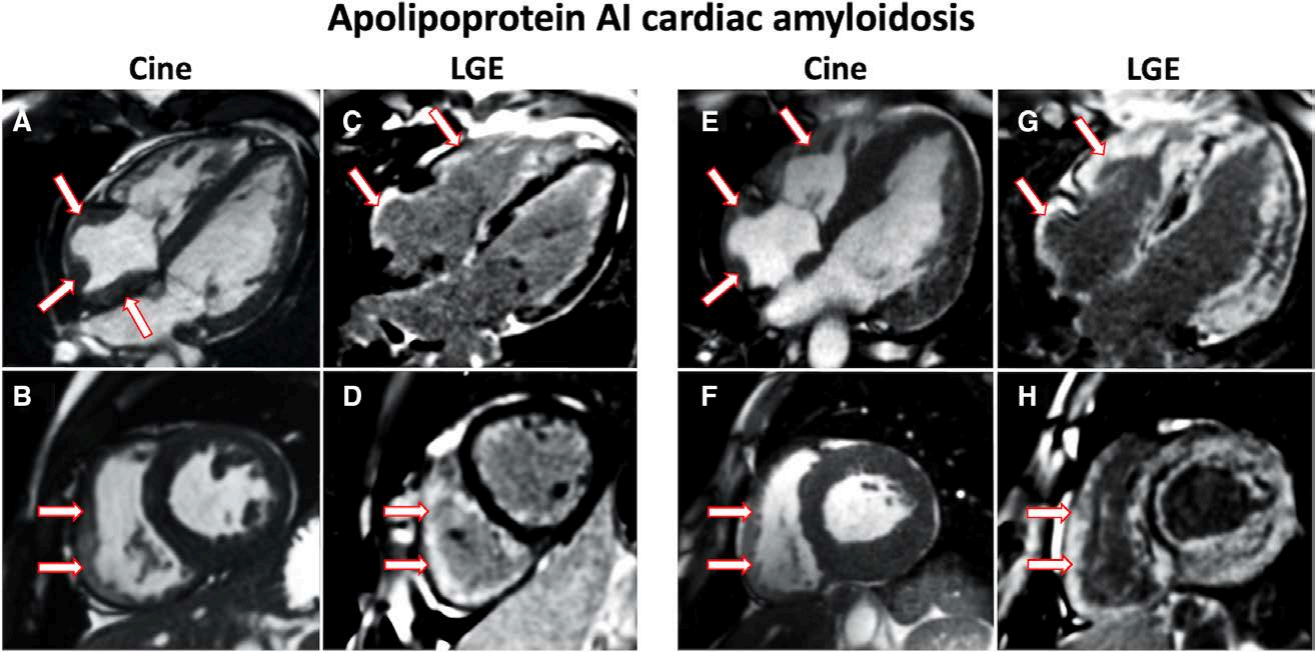
**Cardiac magnetic resonance images of patients with Apo AI cardiac amyloidosis. A**, 4-chamber cine image acquired with steady-state free precession sequence demonstrating prominent right atrial thickening (arrows). **B**, Short axis cine image demonstrating prominent right ventricular thickening (arrows). **C**, 4-chamber late gadolinium enhancement (LGE) image acquired using phase-sensitive inversion recovery sequence reconstructions with steady-state free precession read-outs, demonstrating prominent right atrial and right ventricular LGE (arrows). **D**, Short axis LGE image demonstrating prominent right ventricular LGE (arrows). **E**, 4-chamber cine image demonstrating prominent right atrial (arrows) and right ventricular thickening (arrows). **F**, Short axis cine image demonstrating prominent right ventricular thickening (arrows). **G**, 4-chamber LGE image demonstrating prominent right atrial and right ventricular LGE (arrows). **H**, Short axis LGE image demonstrating prominent right ventricular LGE (arrows).

At diagnosis 6 patients with AApoAI-CA underwent CMR assessment, all of whom had prominent RA and RV thickening, RV LGE and a raised ECV. Patients with cardiac involvement had either of early features of amyloid infiltration (n=4), with normal LV structure and function, but abnormal tissue characterization; or characteristic features of CA (n=2) with increased LV mass, diffuse subendocardial/transmural LGE, and raised ECV (Table S1; Figure 2).

### Apo AIV Amyloidosis

#### Baseline Characteristics

We identified 21 patients with AApoAIV (mean age, 70.9±8.3 years; male, 18 [85.7%]). The following *ApoAIV*-gene variants were identified: Ala161Ser=4, Val13Met=1, Val120Gly=1.

The most common reason for referral was for investigation of heart failure symptoms and local cardiac imaging suggestive of CA (n=12, 57.1%), followed by investigation of chronic kidney disease (n=8, 38.1%) and an incidental finding of amyloidosis on a duodenal biopsy (n=1, 4.8%). AApoAIV commonly presented with cardiac involvement (n=15, 71.4%) and chronic kidney disease stage 3-5 (n=19, 90.5%), of which only 1 patient had nephrotic syndrome. All patients who underwent a renal biopsy had medullary amyloid infiltration with very characteristic morphology. However, due to the absence of proteinuria in the majority of patients, it is unclear whether chronic kidney disease, which was almost universal, was due to renal amyloid infiltration or secondary to heart failure and subsequent renal hypoperfusion. None of the patients in our cohort had liver or spleen involvement, peripheral neuropathy, or a family history of amyloidosis. When compared with AApoAI, patients with AApoAIV had a significantly lower prevalence of liver involvement (0 [0.0%] versus 27 [60.0%], *P*<0.001), spleen involvement (0 [0.0%] versus 28 [62.2%], *P*<0.001) and family history of amyloidosis (0 [0.0%] versus 26 [57.8%], *P*<0.001).

#### Cardiac Phenotype

A total of 15 patients had cardiac involvement, and cardiac involvement was more common than in AApoAI (15 [71.1%] versus 13 [28.9%], *P*=0.001). The most common reason for referral was for investigation of heart failure symptoms and local cardiac imaging suggestive of CA (n=12, 80.0%), and 3 (20.0%) were referred for investigation of chronic kidney disease. Patients with AApoAIV-CA had a significantly lower median estimated glomerular filtration rate than matched patients with AL-CA and ATTR-CA (36 mL/[min·1.73 m²] versus 65 mL/[min·1.73 m²] versus 63 mL/[min·1.73 m²], *P*<0.001).

The 12-lead ECG was available in 14 patients and demonstrated sinus rhythm in half of the patients (n=7) (one of which had first-degree heart block), 6 had atrial fibrillation, and 1 had a ventricular paced rhythm. Only 1 patient had QRS complexes that met the low voltage criteria. All remaining patients had a normal QRS amplitude (limb leads: 0.80±0.17mV; precordial leads: 1.65±0.46 mV), 1 had right bundle branch block, and 1 had left bundle branch block.

At diagnosis, all patients with AApoAIV-CA underwent echocardiographic assessment. All 15 (100.0%) patients had a characteristic echocardiogram, with increased biventricular wall thickness, increased RWT, diastolic dysfunction, impaired longitudinal strain, and a typical apical-sparing strain pattern. There were no significant differences in echocardiographic parameters when compared with matched patients with AL-CA and ATTR-CA. However, when compared with patients with AApoAI-CA, those with AApoAIV-CA more commonly had an apical-sparing strain pattern (15 [100.0%] versus 7 [53.8%], *P*=0.003) and an echocardiogram characteristic of CA (15 [100.0%] versus 5 [61.2%], *P*<0.001).

**Figure 3.**
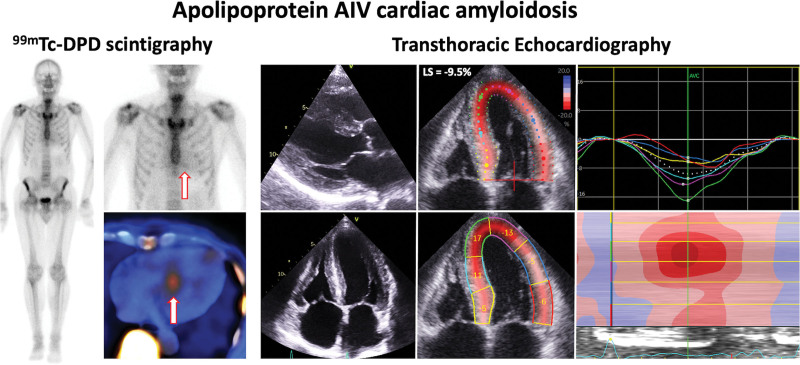
**^99m^Technetium labeled 3,3-diphosphono-1,2-propanodicarboxylic acid (^99m^Tc-DPD) scintigraphy and echocardiographic images in Apo AIV cardiac amyloidosis.** ^99m^Tc-DPD scintigraphy demonstrating grade 1 cardiac uptake and single-photon emission computed tomography demonstrating uptake in the septum (arrows). Transthoracic echocardiography images demonstrating increased biventricular wall thickness, impaired longitudinal strain, and a typical apical-sparing strain pattern with an increased septal apex-to-base strain ratio measured in the 4-chamber view. Images were acquired using a GE Vivid machine, and the strain assessment was carried out using Q-analysis on EchoPac.

At diagnosis 14 patients with AApoAIV-CA underwent ^99m^Tc-DPD scintigraphy, which demonstrated no cardiac uptake in 13 patients and grade 1 myocardial tracer uptake in 1 patient. One patient who had no cardiac uptake on ^99m^Tc-DPD scintigraphy had a repeat scan 4 years later that demonstrated no cardiac uptake, and a third scan 6 years later which demonstrated grade 1 cardiac uptake. Both patients with ^99m^Tc-DPD cardiac uptake had uptake predominantly in the septum (Figure 3). When compared with matched patients with ATTR-CA, those with AApoAIV-CA more commonly demonstrated absent or grade 1 cardiac uptake (14 [100.0%] versus 0 [0.0%], *P*<0.001), whereas patients with AApoAI-CA more commonly demonstrated grade 1 cardiac uptake than patients with AApoAIV-CA (9 [81.8%] versus 2 [14.3%], *P*<0.001).

**Figure 4.**
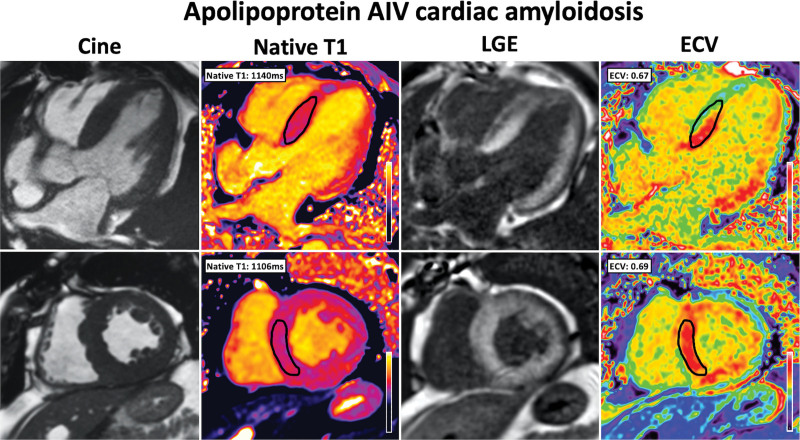
**Cardiac magnetic resonance images in Apo AIV cardiac amyloidosis demonstrate classical features of cardiac amyloidosis.** Cine images were acquired with steady-state free precession (SSFP) sequence, and demonstrate increased wall thickness and left ventricular mass. Native-T1 mapping was acquired using the modified look-locker inversion (MOLLI) recovery sequence and demonstrated an elevated native-T1. Late gadolinium enhancement (LGE) imaging was acquired using phase-sensitive inversion recovery sequence reconstructions with SSFP read-outs and demonstrated diffuse biventricular transmural LGE. T1 mapping was repeated 15 minutes postcontrast using the same slice locations with the MOLLI sequence to produce automated inline extracellular volume (ECV) mapping reconstruction and demonstrated an elevated ECV.

At diagnosis, 9 patients with AApoAIV-CA underwent CMR assessment, all of which had a CMR with characteristic findings of CA. All 9 patients had an increased maximal wall thickness, increased LV mass, and abnormal tissue characterization with LV LGE and an increased ECV (Table 1; Figure 4). There were no significant differences in CMR parameters, when compared with matched patients with AL-CA and ATTR-CA. However, when compared with patients with AApoAI-CA, those with AApoAIV-CA more commonly had a CMR with characteristic findings of CA (9 [100.0%] versus 2 [33.3%], *P*=0.004; Table S1).

**Table 1.**
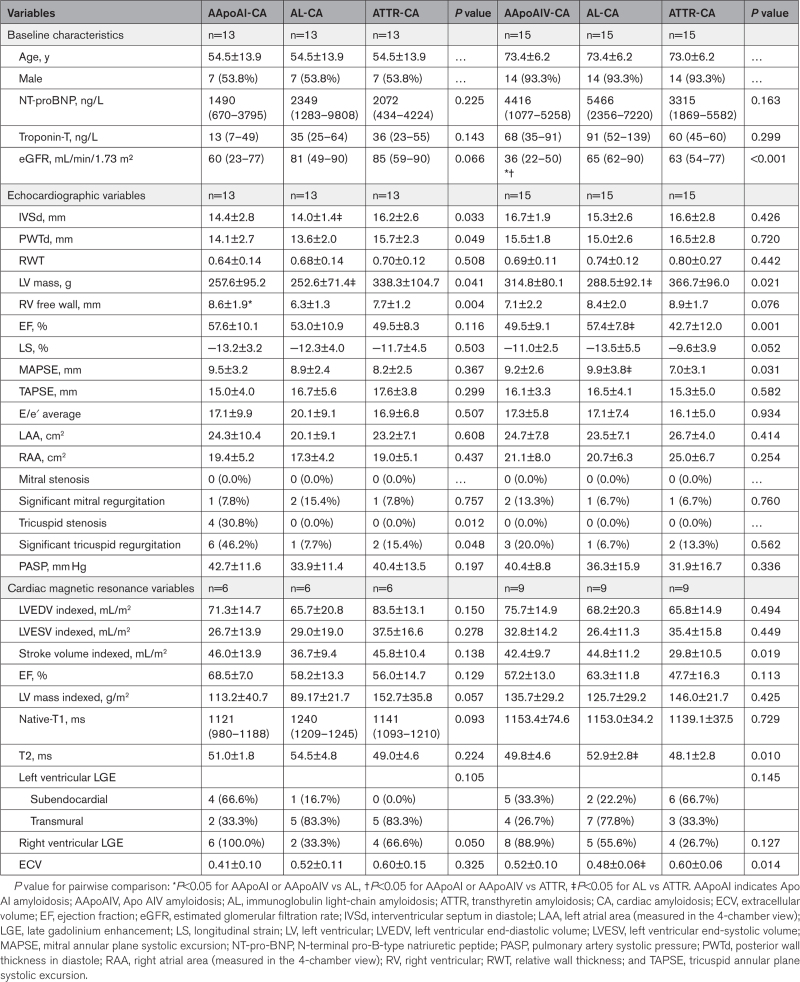
Comparison of Biomarkers and Echocardiographic Parameters Between Patients With AApoAI With Cardiac Involvement and Patients With AApoAVI With Cardiac Involvement

### Survival

**Figure 5.**
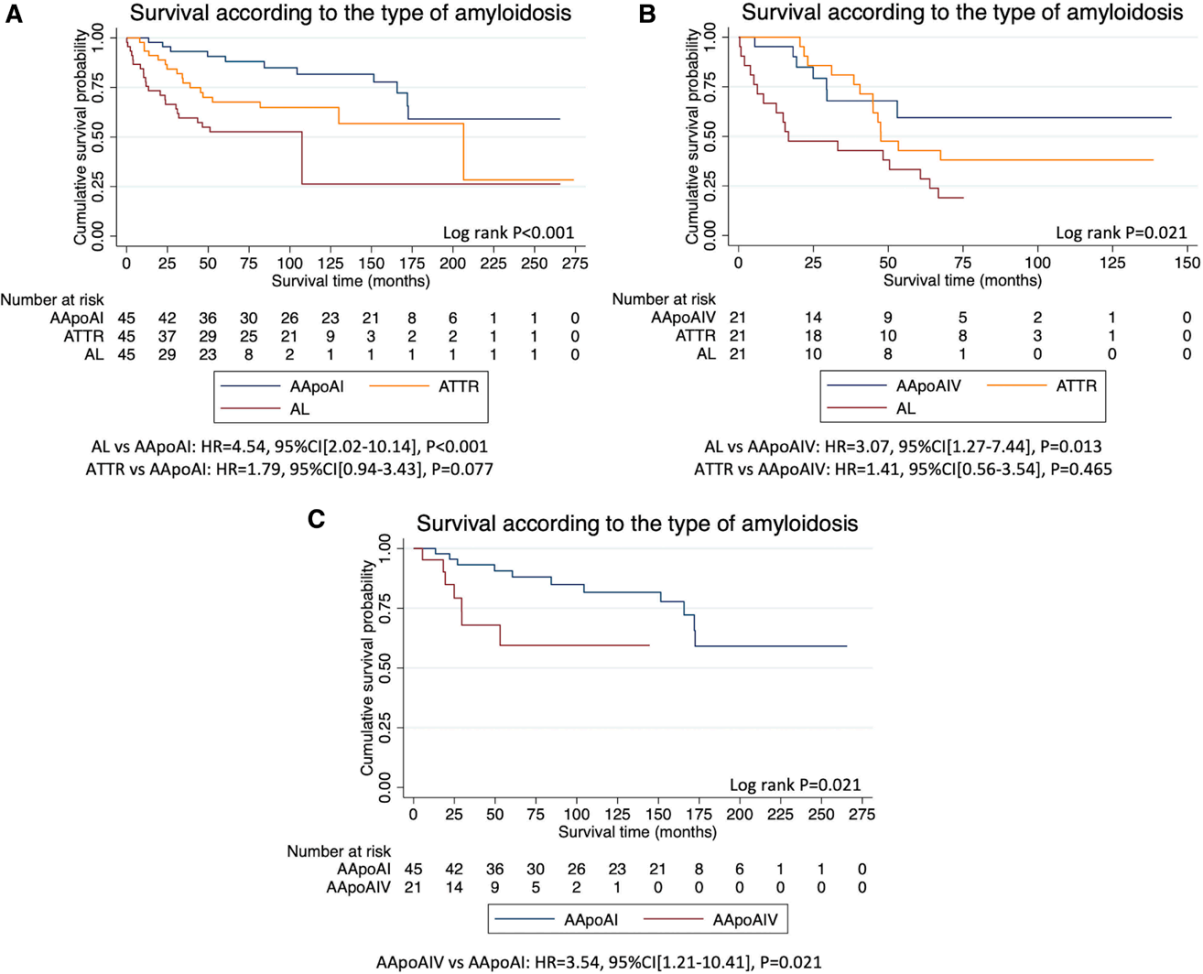
**Kaplan-Meier curves demonstrating survival in different types of amyloidosis. A**, Kaplan-Meier curve comparing survival in patients with Apo AI amyloidosis (AApoAI), to age, sex and cardiac involvement matched patients with transthyretin amyloidosis (ATTR) and immunoglobulin light-chain amyloidosis (AL). **B**, Kaplan-Meier curve comparing survival in patients with apo AIV amyloidosis (AApoAIV), to age, sex and cardiac involvement matched patients with ATTR and AL-amyloidosis. **C**, Kaplan-Meier curve comparing survival in patients with AApoAI and AApoAIV.

Patients with AApoAI had a median follow-up of 133 months (limits of interquartile range, 61–172). Median survival was >172 months (lower 95% CI, 172 months), during which 11 patients died, 5 of whom had evidence of cardiac involvement. However, there was no difference in survival between patients with cardiac involvement and those without cardiac involvement (Figure S1A). Univariable Cox regression analysis revealed that NT-proBNP (HR, 3.46 [95% CI, 1.48–8.05]; *P*=0.004), LV mass (HR, 1.01 [95% CI, 1.00–1.01]; *P*=0.025), biatrial size (left atrial area: HR, 1.08 [95% CI, 1.02–1.13]; *P*=0.006, right atrial area: HR, 1.12 [95% CI, 1.03–1.24]; *P*=0.012), LV ejection fraction (HR, 0.88 [95% CI, 0.80–0.96]; *P*=0.007), longitudinal strain (HR, 1.21 [95% CI, 1.02–1.43]; *P*=0.025) and pulmonary artery systolic pressure (HR, 1.09 [95% CI, 1.02–1.16]; *P*=0.015), were all predictors of mortality. Survival was compared to a cohort of 45 patients with AL-amyloidosis and 45 patients with ATTR-amyloidosis who were matched for age, sex, and presence or absence of cardiac involvement. Patients with AApoAI had a reduced risk of mortality compared with patients with AL-amyloidosis, but there was no significant difference when compared to matched patients with ATTR-amyloidosis (Figure 5A).

Patients with AApoAIV had a median follow-up of 42 months (limits of interquartile range, 21–61). Median survival was >30 months (lower 95% CI, 30 months), during which 7 patients had died, all of whom had evidence of cardiac involvement. Patients with cardiac involvement had a statistically significant reduction in survival compared with those without cardiac involvement (log-rank *P*=0.039; Figure S1B). Univariable Cox regression revealed that NT-proBNP (HR, 5.94 [95% CI, 1.11–31.74]; *P*=0.037), interventricular septal diameter (HR, 1.37 [95% CI, 1.04–1.81]; *P*=0.028), LV mass (HR, 1.01 [95% CI, 1.00–1.02]; *P*=0.022), LV ejection fraction (HR, 0.94 [95% CI, 0.89–0.99]; *P*=0.028), and longitudinal strain (HR, 1.28 [95% CI, 1.01–1.61]; *P*=0.038) were all predictors of mortality. Survival was compared with a cohort of 21 patients with AL-amyloidosis and 21 patients with ATTR-amyloidosis who were matched for age, sex, and presence or absence of cardiac involvement. Patients with AApoAIV had a reduced risk of mortality compared with patients with AL-amyloidosis, but there was no significant difference when compared with matched patients with ATTR-amyloidosis (Figure 5B). When compared with the 45 patients with AApoAI, the 21 patients with AApoAIV had an increased risk of mortality (Figure 5C).

## DISCUSSION

This is the first study to extensively characterize the cardiac phenotype in patients with AApoAI and AApoAIV. Our study has demonstrated that: (1) AApoAI is commonly associated with multisystemic disease, and cardiac involvement presented with heart failure or dysphonia; (2) the Arg173Pro variant was always associated with both cardiac and laryngeal involvement; (3) AApoAI-CA commonly displays right-sided involvement, with RV thickening, tricuspid valve dysfunction, RA and RV radiotracer uptake, and RA and RV LGE; (4) AApoAIV presented with cardiac or renal involvement; (5) AApoAIV-CA commonly presented with heart failure symptoms and always displayed classical features of CA on echocardiography and CMR, hence mimicking more common forms of CA; (6) both AApoAI and AApoAIV are associated with a good prognosis and a lower risk of morality than matched patients with AL-amyloidosis.

AApoAI is commonly associated with a strong family history of amyloidosis and multisystemic disease resulting in renal, splenic, hepatic, cardiac, laryngeal, nerve, and cutaneous involvement. Therefore, CA in a patient with a family history of amyloidosis or multisystemic involvement, especially when associated with dysphonia, should raise the suspicion of AApoAI. Cardiac involvement appears to be highly dependent on the genetic variant and was most commonly associated with the Arg173Pro variant. This variant was also strongly associated with laryngeal involvement and in some cases associated with a petechial rash.^17^ Our data confirm a strong association between the Arg173Pro variant and cardiac involvement and highlight the need to fully assess the degree of cardiac infiltration if the Arg173Pro variant is identified. Furthermore, the presence of dysphonia or a petechial rash in a patient with imaging features of CA should prompt consideration of AApoAI within the differentials.

**Figure 6.**
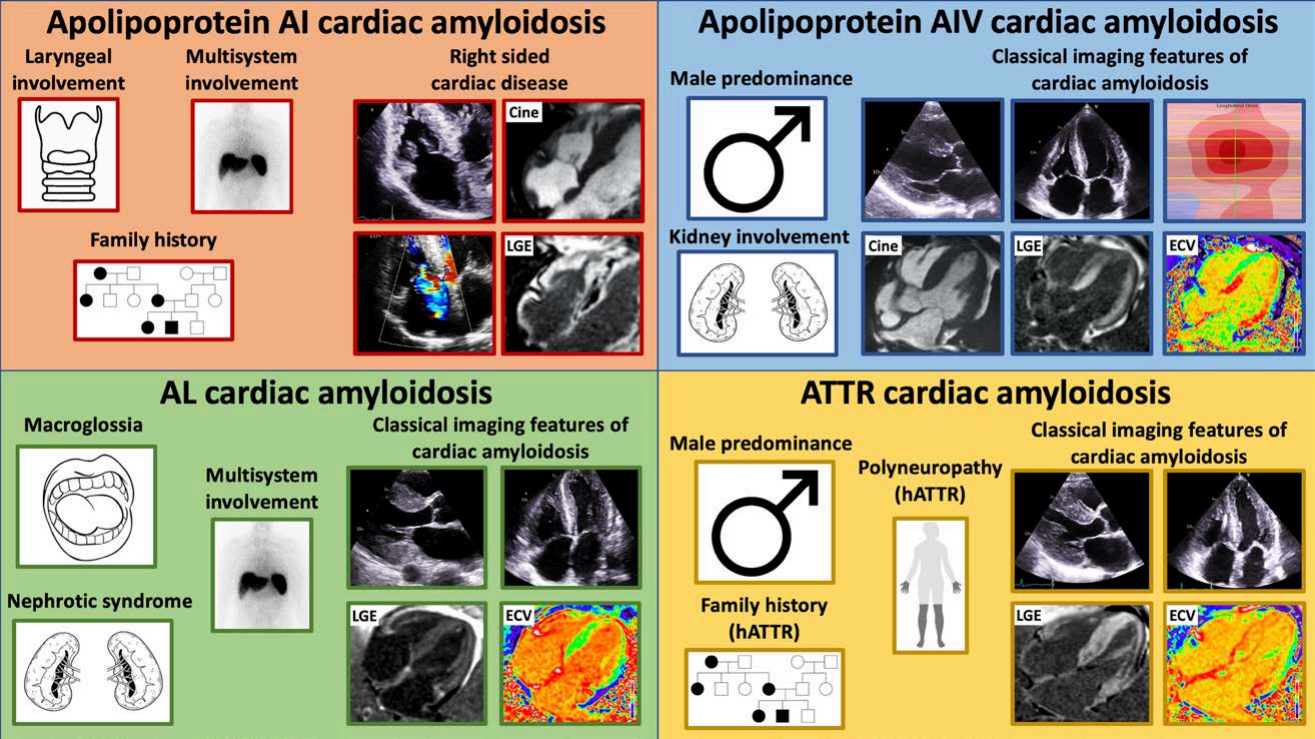
**Diagram illustrating key features in the clinical history and on cardiac imaging that should raise the suspicion of the different forms of cardiac amyloidosis. Top left**, Apo AI cardiac amyloidosis can present with laryngeal involvement, multiorgan involvement, and a strong family history. Echocardiographic images demonstrate right-sided disease with thickening of the tricuspid valve and tricuspid regurgitation. Cardiac magnetic resonance (CMR) demonstrates right atrial and right ventricular thickening, and right atrial and right ventricular late gadolinium enhancement (LGE). **Top right**, Apo AIV cardiac amyloidosis has a male predominance and can present with renal involvement. Echocardiographic images demonstrate biventricular wall thickening and a typical apical-sparing strain pattern. CMR demonstrates left ventricular wall thickening, biventricular transmural LGE, and an elevated extracellular volume (ECV). **Bottom left**, Immunoglobulin light-chain (AL) cardiac amyloidosis can present with macroglossia, multisystem involvement, and nephrotic syndrome. Echocardiographic images demonstrate biventricular wall thickening. CMR demonstrates diffuse biventricular transmural LGE and an elevated ECV. **Bottom right**, Transthyretin (ATTR) cardiac amyloidosis has a male predominance and can present with polyneuropathy and a strong family history. Echocardiographic images demonstrate biventricular wall thickening. CMR demonstrates diffuse biventricular transmural LGE and an elevated ECV. hATTR indicates hereditary ATTR.

Following extensive characterization with multimodality imaging, we identified distinctive features of right-sided involvement with RA thickening, RV thickening, and tricuspid valve dysfunction seen on echocardiography. The prominent right-sided cardiac disease was evident on bone scintigraphy with RA and RV radiotracer uptake; and CMR which demonstrated prominent RA and RV thickening, alongside marked RA and RV LGE. Patients had significant valvular involvement, with thickening of the tricuspid valve leaflets and subvalvular apparatus, tethering of the septal leaflet leading to significant tricuspid regurgitation or stenosis. The degree of RV thickening and prevalence of tricuspid valve dysfunction was significantly greater than in matched patients with ATTR-CA and AL-CA. AApoAI-CA can produce a spectrum of imaging findings, with some patients having classical imaging features of CA, whereas the majority presented with right-sided cardiac disease. Underappreciation of this rare form of CA among clinicians could result in patients being incorrectly labeled as having one of the more prevalent forms of CA (ATTR or AL). Identification of key aspects in the clinical presentation such as a family history of amyloidosis, multisystemic disease, dysphonia, or identification of right-sided cardiac disease on cardiac imaging should prompt consideration of AApoAI within the differentials and subsequent genetic screening (Table 2; Figure 6).

**Table 2.**
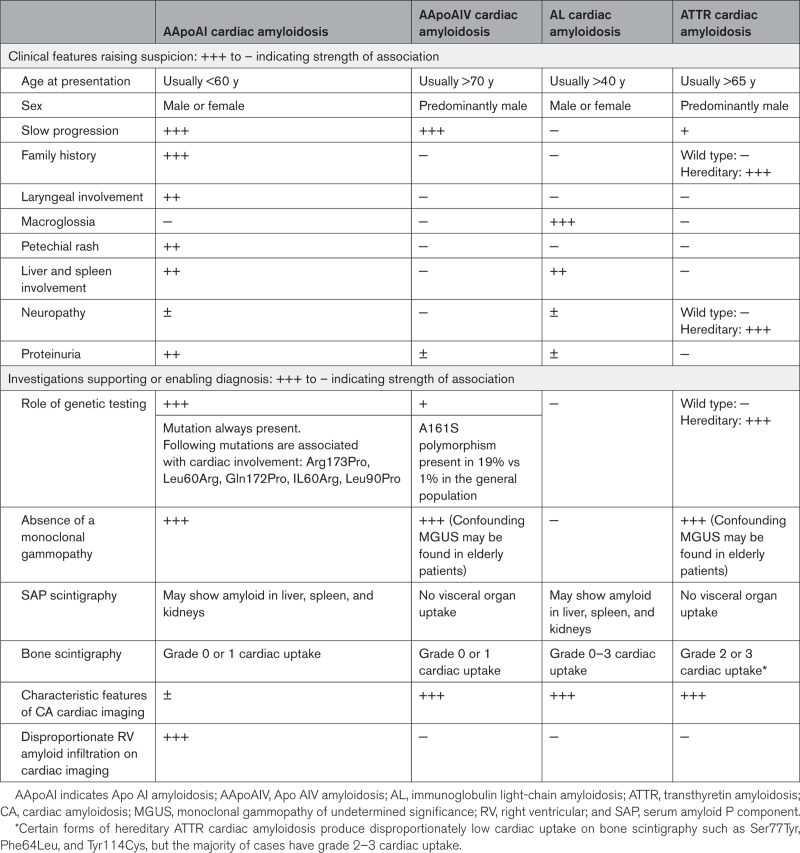
Summary of the Clinical Features That Should Raise the Suspicion of AApoAI, AApoAIV, AL, and ATTR, and a Summary of the Supporting Diagnostic Tests

AApoAIV is associated with cardiac or renal involvement. Cardiac involvement presented with symptoms of heart failure and always produces classical features of CA on both echocardiography and CMR. Up until now, it was thought that patients with AApoAIV-CA did not have any cardiac radiotracer uptake on bone scintigraphy, and hence the presence of cardiac uptake may have been used clinically to exclude AApoAIV-CA from the diagnostic differentials.^18^ However, we have identified 2 cases of AApoAIV-CA that resulted in grade 1 cardiac uptake on bone scintigraphy. Considering the cardiac phenotype of patients with AApoAIV-CA, there is a considerable overlap with AL-CA, and hereditary ATTR-CA variants, such as Ser77Tyr, Tyr114Cys and Phe64Leu,^19–21^ which can all present with heart failure symptoms, classical features of CA on echocardiography/CMR, but a disproportionately low cardiac radiotracer uptake on bone scintigraphy (ie, grade 0 or 1). This is further supported by the similarities seen on echocardiography and CMR between patients with AApoAIV-CA and matched patients with ATTR-CA and AL-CA. The diagnostic pathway for cardiac radiotracer uptake grade 2 to 3 is well established,^22^ but grade 0 to 1 still present a diagnostic challenge.^21^ This is further complicated by the knowledge that AApoAIV-CA can mimic the phenotype of more common forms of CA (Table 2; Figure 6).

Suspicion of either rare form of CA should be prompted in patients with either characteristic features of CA, or right-sided cardiac disease on cardiac imaging, in association with grade 0 or 1 cardiac uptake on bone scintigraphy, and the absence of biochemical evidence of a plasma cell dyscrasia (although it is noteworthy that elderly patients may have a monoclonal gammopathy of undetermined significance).^23^ The low-grade or absent cardiac radiotracer uptake in AApoAI-CA and AApoAIV-CA is not proportional to the degree of cardiac infiltration, and therefore, standalone bone scintigraphy cannot be used to diagnose these rarer forms of CA. Instead, a multi-imaging diagnostic approach, combined with both genetic and histological analysis is required to confirm a diagnosis. Our findings highlight the importance of assessing for a plasma cell dyscrasia in all patients and underscore the need for obtaining a histological diagnosis in patients with grade 0 to 1 cardiac uptake on bone scintigraphy, not only to discover rarer forms of CA but also to exclude systemic AL-amyloidosis, which requires cytotoxic chemotherapy-based treatments,^3^ and ATTR for which highly specific and costly disease-modifying treatments are available.^8,9^

In contrast to more prevalent forms of CA, the treatment for AApoAI and AApoAIV is largely supportive, with the mainstay of medical therapy revolving around managing complications of the disease process. There have been reports of organ regression in AApoAI following successful liver transplantation, due to a reduction in hepatic synthesis of the ApoAI-amyloid protein. However, given the significant morbidity and mortality associated with liver transplantation, this treatment option remains reserved for selected cases.^24^

Cardiac involvement is regarded as the main driver of mortality in amyloidosis, although to date this has mainly been demonstrated in patients with more common forms of amyloidosis, namely systemic AL-amyloidosis and ATTR.^1,2^ Our study has demonstrated that in both AApoAI and AApoAIV, increased NT-proBNP, LV mass, biatrial size, and longitudinal strain were all associated with an increased risk of mortality. Despite these observations, both AApoAI and AApoAIV are slowly progressive and carry a good prognosis.

### Limitations

The main limitation of our study is that it is a single-center study with a small sample size, but this reflects the rare nature of these diseases. Acquisition of the RV-focused view required for RV strain measurements was not part of our standard echocardiographic protocol, but considering the right-sided cardiac disease in AApoAI-CA, this could be considered in future studies. CMR imaging was only available in some patients within our cohort who had a clinical indication for a CMR at the time of diagnosis. The small sample size meant that our survival analysis was limited to univariable Cox regression, and underpowered for a multivariable analysis.

### Conclusions

In summary, AApoAI-CA commonly presents with heart failure symptoms or dysphonia, and right-sided cardiac disease. AApoAIV-CA commonly presents with heart failure symptoms and always displays characteristic features of CA on echocardiography/CMR. Appreciation of not only the unique characteristic features of AApoAI but also the capability of AApoAIV to mimic common forms of CA is urgently needed in clinical practice to reduce the risk of misdiagnosis. Finally, both AApoAI and AApoAIV are slowly progressive and associated with a good prognosis.

## ARTICLE INFORMATION

### Sources of Funding

Dr Fontana is supported by a British Heart Foundation (BHF) Intermediate Clinical Research Fellowship (FS/18/21/33447). Daniel Knight is supported by a BHF Clinical Research Leave Fellowship (FS/CRLF/20/23004).

### Disclosures

Dr Fontana has consulting income from Intellia, Novo-Nordisk, Pfizer, Eidos, Prothena, Alnylam, Alexion, Janssen, and Ionis. Dr Gillmore has consulting income from Ionis, Alexion, Eidos, Intellia, Alnylam, and Pfizer. A. Wechelakar has consulting income from Alexia, Astra-Zeneca, Janssen, Attralus, and Prothena. Dr Hawkins has consulting income from Alnylam. The other authors report no conflicts.

### Supplemental Material

Supplemental Methods

Biopsy Data

Table S1

Figure S1

## Supplementary Material
