# Supplementary material for: Rare Forms of Cardiac Amyloidosis: Diagnostic Clues and Phenotype in Apo AI and AIV Amyloidosis
Source: Circ Cardiovasc Imaging. 2023 Jul 11;16(7):523–35. doi: 10.1161/CIRCIMAGING.123.015259 (PMC10351905; doi:10.1161/CIRCIMAGING.123.015259)
Supplement: Supplementary file 1 [file hci-16-523-s001.pdf]

SUPPLEMENTAL MATERIAL

Supplemental methods 2
^{99m}Tc-DPD Bone Scintigraphy Protocol and Image Analysis 2
SAP scintigraphy 2
Echocardiography 2
Cardiac Magnetic Resonance Imaging 3
Biopsy data 4
AApoAI 4
AApoAIV 4
Supplementary Table S1 5
Supplementary Figure S1 6

Supplemental methods

^{99m}Tc-DPD Bone Scintigraphy Protocol and Image Analysis

Images were acquired by using the clinical SPECT/CT hybrid camera (Discovery NM/CT 670, GE Healthcare, Haifa, Israel). Patients were intravenously injected with approximately 700 MBq (18.9 mCi) of ^{99m}Technetium labelled 3,3-diphosphono-1,2-propanodicarboxylic acid (^{99m}Tc-DPD). The protocol consisted of planar whole-body acquisition performed at 3-hours post injection, followed by single-photon emission computed tomography (SPECT) imaging of the thorax. A low-dose, non-contrast CT scan was performed for attenuation correction and anatomic localization, generating a volume of interest for the whole heart. RV involvement was defined as RV radiotracer uptake.

SAP scintigraphy

Serum amyloid P component (SAP) scintigraphy was utilised to assess for visceral organ amyloid infiltration. Anterior and posterior whole-body images were acquired following ^{123}I-SAP administration using a General Electric Infinia Hawkeye (Hybrid NM/CT, GE Healthcare, Milwaukee, USA) or Discovery 670 Gamma Camera (Discovery NM/CT, GE Healthcare, Milwaukee, USA) with extended low-energy general-purpose collimators. Visceral amyloid burden was scored by visual assessment.

Echocardiography

All echocardiograms were performed using a GE Vivid machines (GE Logiq E9, GE Healthcare, Solingen, Germany) and analysed offline using the most up to date EchoPAC software at the time of image acquisition. RV involvement was defined as an RV free wall thickness >5mm, and valvular regurgitation was deemed significant if at least moderate or severe.

Cardiac Magnetic Resonance Imaging

All CMR scans utilised a 1.5-T clinical scanner (Magnetom Aera, Siemens Healthineers, Erlangen, Germany). Within a conventional clinical scan (localizers and cine imaging with steady state free precession (SSFP) sequence), LGE imaging was acquired with both magnitude inversion recovery (MAG-IR) and phase-sensitive inversion recovery (PSIR) sequence reconstructions with SSFP read-outs. T1 measurement was performed with the use of the modified look-locker inversion (MOLLI) recovery sequence. After a bolus of gadoterate meglumine (0.1 mmol/kg, gadolinium-DOTA, Dotarem, Guerbet S.A. France) and LGE imaging, T1 mapping was repeated 15-minutes post-contrast using the same slice locations with the modified look-locker inversion recovery sequence, to produce automated inline ECV mapping reconstruction. T1-mapping protocols used 5s(3s)3s and 4s(1s)3s(1s)2s sampling, pre- and post-contrast, respectively.

Biopsy data

AApoAI

Diagnosis was confirmed though identifying a pathogenic gene variant in all patients, while histological diagnosis was confirmed in 28(62.2%) patients. The most common biopsy sites were kidney (AApoAI identified in 11/12) and liver (AApoAI identified in 8/8). Less common biopsy sites were, laryngeal (AApoAI identified in 4/4), cardiac (AApoAI identified in 2/2) and duodenal (AApoAI identified in 1/1). Ethnicity data was available for 42 patients, of which 24(57.1%) were White British, 13(31%) White Irish, 4(9.5%) White Other and 1(2.3%) Indian.

AApoAIV

The most common biopsy sites were cardiac (AApoAIV identified in 13/13) and renal (AApoAIV identified in 7/8). Less common biopsy sites were bone marrow (AApoAIV identified in 2/3), duodenal (AApoAIV identified in 1/1) and bladder (AApoAIV identified in 1/1). Ethnicity data was available for 18 patients, of whom 16 were White British and 2 White Irish.

Supplementary Table S1

**12 lead resting electrocardiogram findings**	**AApoAI-CA (n = 13)**	**AApoAIV-CA (n = 14)**	**P-value**
Sinus rhythm	9 (69.2%)	7 (50.0%)	0.310
Atrial fibrillation	2 (15.4%)	6 (42.9%)	0.118
Small QRS complexes	2 (15.4%)	1 (7.1%)	0.496
Ventricular paces rhythm	2 (15.4%)	1 (7.1%)	0.496
**Echocardiographic findings**	**AApoAI-CA (n = 13)**	**AApoAIV-CA (n = 15)**	**P-value**
Echocardiogram suggestive of cardiac amyloidosis	8 (38.5%)	0 (0.0%)	**<0.001**
Echocardiogram characteristic of cardiac amyloidosis	5 (61.2%)	15 (100.0%)	**<0.001**
'Apical sparing' strain pattern	7 (53.4%)	15 (100.0%)	**0.003**
Mitral stenosis	0 (0.0%)	0 (0.0%)	-
Significant mitral regurgitation	1 (7.7%)	2 (13.3%)	0.630
Tricuspid stenosis	4 (30.8%)	0 (0.0%)	**0.023**
Significant tricuspid regurgitation	6 (46.2%)	3 (20.0%)	0.139
**Bone scintigraphy**	**AApoAI-CA (n = 11)**	**AApoAIV-CA (n = 14)**	**P-value**
Grade 0 cardiac uptake	4 (36.4%)	13 (92.9%)	**0.003**
Grade 1 cardiac uptake	7 (63.6%)	1 (7.1%)	**0.003**
Grade 2 cardiac uptake	0 (0.0%)	0 (0.0%)	-
Grade 3 cardiac uptake	0 (0.0%)	0 (0.0%)	-
**CMR findings**	**AApoAI-CA (n = 6)**	**AApoAIV-CA (n = 9)**	**P-value**
Early features of cardiac amyloid infiltration	4 (66.7%)	0 (0.0%)	**0.004**
Characteristic features of cardiac amyloid infiltration	2 (33.3%)	9 (100.0%)	**0.004**
Subendocardial LV LGE	4 (66.7%)	5 (55.6%)	0.667
Transmural LV LGE	2 (33.3%)	4 (44.4%)	0.667
RV LGE	6 (100.0%)	8 (88.9%)	0.398

Supplementary Table S1. Summary of the cardiac phenotype at diagnosis of patients with apolipoprotein AI cardiac amyloidosis (AApoAI-CA) and apolipoprotein AVI cardiac amyloidosis (AApoAIV-CA). LV = Left ventricular; LGE = Late gadolinium enhancement; RV = Right ventricular.

Supplementary Figure S1

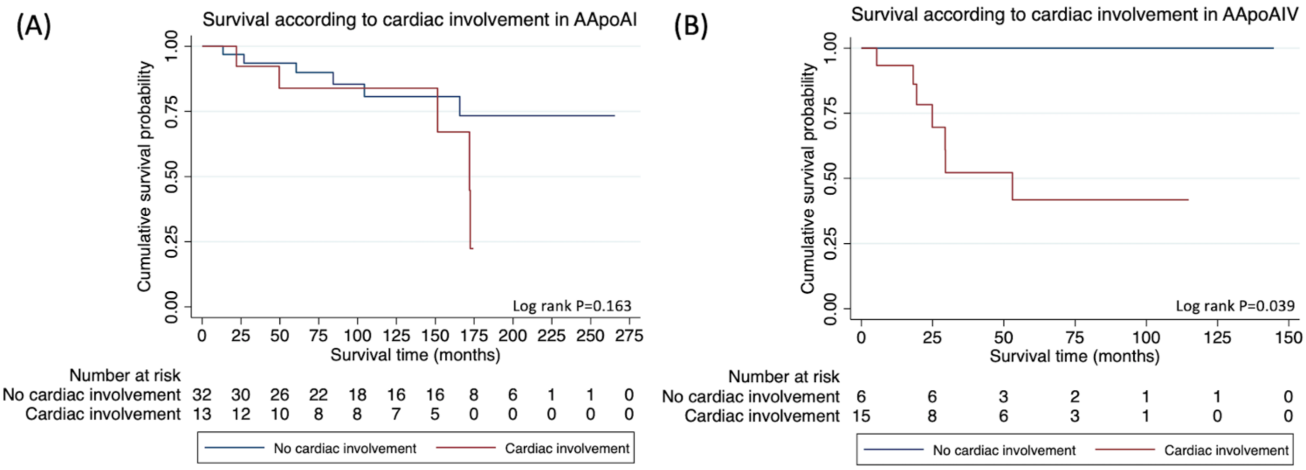

Supplementary Figure S1. Kaplan-Meier curves comparing survival in patients with cardiac involvement to those without cardiac involvement.

(A) Kaplan-Meier curve of patients with apolipoprotein AI amyloidosis (AApoAI) comparing survival in those cardiac involvement to those without cardiac involvement.

(B) Kaplan-Meier curve of patients with apolipoprotein AIV amyloidosis (AApoAIV) comparing survival in those cardiac involvement to those without cardiac involvement.
